# What good is anthropology? Care work in a “useless” discipline

**DOI:** 10.1111/amet.13253

**Published:** 2023-12-27

**Authors:** Nolwazi Mkhwanazi

**Affiliations:** ^1^ Centre for the Advancement of Scholarship University of Pretoria

**Keywords:** anthropology, care, care work, South Africa

## Abstract

Different forms of care work are essential for the practice of anthropology in South Africa. In this biographical commentary, I describe how I enacted care work in my anthropological practice. I suggest that what is good about anthropology is its potential to be attentive to the multiple ways in which care work is enacted by us as anthropologists, as teachers of the discipline, as well as by our interlocutors.

## LEAVING ANTHROPOLOGY TO RETURN AGAIN

Several times, over the past 15 years, I have tried to leave the discipline of anthropology. Yet I have always found myself returning to it because anthropology holds the promise of allowing us to do some good in a world that is increasingly polarized, unjust, unequal, and unfair. Care work, I suggest, is central to the potential good of anthropology.

The first time I left anthropology was in 2010, when I resigned from my tenured position at the East London campus of the University of Fort Hare, a historically Black institution. The university is well known for its role in educating Black people during the apartheid era, a time when the provision of education for people who were not classified as white was given little importance and deliberately underresourced.

Apartheid (Afrikaans for “apartness”) was legalized racism. As noted by Graham Pechey (2005, p. 209), the late writer and anti‐apartheid activist, this word signifies “brutally binary habits of perception.” While racial segregation had existed before under British colonial rule, when the Afrikaner National Party came to power in 1948, it introduced a series of acts that aimed to strengthen white minority rule through the “‘maintenance and protection’ of Afrikanerdom, white power, and the white race” (Beinart, 2001, p. 147). These acts affected all spheres of life for people who resided in South Africa, having an impact on family life and shaping access to and the quality of education, housing, and health care. Apartheid ideology, with its insistence on “separate development” for different races, ensured different realities and possibilities depending on whether one was classified as African, colored, Indian, or white. In a moving Nobel Peace Prize acceptance speech given in Oslo in 1961, Chief Albert J. Luthuli, the then president general of the liberation movement the African National Congress (ANC), described life under apartheid as follows:

Here [in South Africa] the cult of race superiority and of white supremacy is worshipped like a god. Few white people escape corruption and many of their children learn to believe that white men are unquestionably superior, efficient, clever, industrious and capable; that black men are, equally unquestionably, inferior, slothful, stupid, evil and clumsy. On the basis of the mythology that “the lowest amongst them is higher than the highest amongst us,” it is claimed that white men build everything that is worthwhile in the country; its cities, its industries, its mines and its agriculture, and that they alone are thus fitted and entitled as of right to own and control these things, whilst black men are only temporary sojourners in these cities, fitted only for menial labour, and unfit to share political power. (Luthuli, 1987, p. 17)

The introduction of the Extension of Universities Education Act of 1959 prohibited racially integrated education except in extraordinary cases, subject to government approval. Universities became racially segregated and catered to particular linguistic groups—for example, the University of Zululand catered to the education of amaZulu and amaSwati. Through the Extension of Universities Education Act, and additional legislation, the University of Fort Hare was designated as a Black institution for amaXhosa (Subotzky, 1997). Nonetheless, throughout Fort Hare's history, many Black African leaders, politicians, and academics from around the continent have been educated there.^1^

Under apartheid, Black universities were underresourced and had poor infrastructure. Their research outputs and productivity were low (for an explanation of why this was the case, see Mngomezulu, 2020). These deep fractures in university resource allocation, funding, and research opportunities were set in motion during apartheid, and they continue today. Acknowledging ongoing racial and gendered inequalities, South African universities in the postapartheid era have been grappling with transformative agendas in response to apartheid legacies while seeking ways to achieve social redress and equity and to expand participation in higher education.

Fort Hare, now classified as a historically “disadvantaged” university, remains underresourced, and the majority of the student body is still Black. I accepted a position there because I wanted to teach Black students anthropology. Before this, I had been a postdoctoral fellow at the University of Cape Town, a historically white (and advantaged) university that was (and still is) struggling to undergo transformation. My move to Fort Hare felt deeply political and personal. While some of my anthropology colleagues were puzzled by what they saw as a step down in the hierarchy of universities and anthropology departments, I saw my move as an important act of self‐care.

What constitutes care is a complex. Care is an elusive and “unsettling” concept (Cook & Trundle, 2020). It can be loving, positive, creative, and attuned to practices that produce and build relationships that sustain life. It can also be intentionally cruel, violent, and disruptive, engendering negative feelings and the severing of relationships. What constitutes an act of care is not always experienced or perceived as such by others, including the person the act of care is directed toward or even other caregivers (McKearney, 2020). Perceptions of care are imbued with morality, and political and economic considerations—indeed, care is experienced differently according to gender, race, and generation. Furthermore, it is temporal and “attends to the life course and ideas about need, deservingness and care responsibilities” (Thelen, 2015, p. 506).

Having moved to the University of Fort Hare as an act of self‐care, I resigned after three years because of my growing unease with how anthropology was being taught there. I was uncomfortable with the attitude of white colleagues toward the mainly Black student body, who were often treated as “native informants” rather than engaged scholars. Francis Nyamnjoh has similar observations about the relationship between white and Black anthropologists in South Africa. He writes,

So, try as black and coloured anthropologists may to be seen and treated as equals among white anthropologists, local or foreign, they are almost invariably perceived as more “native” or as “the Other”—the very stuff that makes anthropology possible—and therefore cannot claim to practise anthropology; they should be inviting bona fide anthropologists to practise anthropology on them. (Nyamnjoh, 2012, p. 74)

My decision to move away from anthropology meant leaving the university altogether. I took up a job in an NGO that specialized in research on HIV and AIDS in South Africa. In our collaborations with US‐based organizations and funding bodies, we were treated as data collectors incapable of analyzing data or writing reports (a similar point is made by Obbo, 2006, p. 154). Thus, my time at the NGO was short lived.

I returned to anthropology in 2012, accepting a tenured position at the University of the Witwatersrand (Wits), a historically white university in Johannesburg. I was the only tenured staff member who was Black in a department of six people—three of whom were white men and held senior positions. In 2015, two historically white universities were at the center of protests—Wits and the University of Cape Town. The protests, known as #FeesMustFall, were led primarily by Black students. They began as demonstrations against proposed increases on high university fees, which made university education, with the shrinking of state funding, out of reach for many young Black people. As the protests evolved, the list of demands grew: prominent additions were the decolonization of the education system and the transformation of the universities to address racial and gender inequalities—particularly staff composition—as well as the insourcing of general workers (Langa, 2017).

Paying homage to its history of speaking truth to power, the Wits anthropology department provided space for students to meet and strategize. In the process, some of the staff became vocal and visible participants in the protests, supporting the students’ calls for transformation in the university and the decolonization of the curriculum. When the student protests died down, everything returned to business as usual. The work of a few Black (mainly non–South African) scholars was added to the curriculum, but attempts to hire more Black academic staff in a majority‐white anthropology department were contested by the anthropology academic faculty. For months I felt betrayed and heartbroken because I thought that, as anthropologists, we would be more sensitive to the need to change the department's racial and gendered imbalance. In 2017, I accepted a secondment to a multidisciplinary research institute within the university and left the anthropology department. This second attempt to leave anthropology was another act of self‐care.

In certain African countries and even among some of my colleagues in South African universities, anthropology is not well regarded. For example, in 2022 the Kenyan cabinet secretary of education, Professor Magoha, stated that anthropology was a “useless” course and that those wanting to study medicine but who had not qualified should take courses in public health rather than anthropology. Despite having joined and left two anthropology departments and one anthropology research institute during my academic career, I continue to fiercely advocate for the discipline and the importance of ethnographic research. Ethnography exposes us to diverse understandings and experiences of the world. It enables us to witness, make sense of, and explain the varied ways that people inhabit, experience, and make sense of their lives. The good of anthropology is in enriching the global thoughtscape.

The prompt for this essay—What good is anthropology?—came at a time when I wanted to think through my passion for anthropology. I had recently published an article that I felt captured my vexed relationship with the discipline in South Africa (Mkhwanazi, 2023). I am, after all, a practitioner of a discipline that is, in many countries on the African continent, viewed by some as “useless” or worse, a remnant of colonial legacy, one that, at least in South Africa, still struggles to embrace local Black anthropological scholarship (on the various and complex attitudes toward anthropology in Africa, and perceptions of what constitutes anthropological knowledge about Africa, see Obbo, 2006). In the remainder of the commentary, I describe how I initially became interested in anthropology. I discuss some of the research that I have done in which attention to care work has been important. I end with some thoughts about care for the discipline and its future practitioners in Africa.

## ANTHROPOLOGY—BIRTH TALES

My introduction to the discipline of anthropology was serendipitous. When I enrolled for my first year at the University of Cape Town in 1995, I had no idea what anthropology was. Looking for a fourth course (having already chosen philosophy, political science, and economics), I chose anthropology because I was told that it was the study of other cultures. I had attended a high school in Eswatini (then Swaziland) that was born out of an anti‐apartheid vision to create a space where racially and culturally diverse students could live and learn together regardless of their financial ability. The school was founded in 1963. It is known for having educated the children and grandchildren of many anti‐apartheid activists, including those of Nelson Mandela, Walter Sisulu, and Archbishop Desmond Tutu. My exposure to the anti‐apartheid movement and anti‐racist ideology, and being educated in a culturally diverse context, informed my decision to study anthropology.

Sitting in a second‐year medical anthropology lecture, I realized that I could also pursue my newfound interest in pregnancy, childbirth, and the work of birthing practitioners through anthropology. I had just finished a gap year in the United States, where I had lived with a midwife, Mary Kroeger, and had become interested in the work of midwives. At a gathering that Mary and I attended in Monroe, Utah, I witnessed women crying when they spoke about how they were treated when they gave birth. I had never given much thought to birth, let alone imagined it as a traumatic event. I wondered what stories women in South Africa would tell, given the country's complex racial dynamics and the apartheid government's racially skewed population policies (Brown, 1987; Chimere‐Dan, 1993). I also wondered if anyone had asked them about their experiences. Looking back, I have come to see that those women's telling of their birth stories in Utah was not only cathartic but also an extension of the care provided by the mainly lay midwives who had gathered with them—sitting with, listening to, and sometimes shedding tears with the mothers.

In my anthropological work, I have been able to listen to women (young and old) speak about the experiences of pregnancy and childbirth. This is important because in South Africa teenage pregnancy is still treated as an abomination. As I write, there is a petition doing the rounds on social media titled “teenage pregnancy is statutory rape.”^2^ While the petition is well intended, my colleagues and I have pointed out, in a letter addressed to the health minister, that it further stigmatizes pregnant teenagers and teenage parents (Macleod et al., 2023). Teenage mothers are often shamed, teased, and taunted by their peers, family members, teachers, and even midwives when giving birth. By asking young women about their experiences of becoming mothers and listening to them, researchers can help them craft narratives that are important to them and their communities at that moment in time.

In my initial research on teenage pregnancy (1999–2001) among Xhosa‐speaking people in urban townships of Cape Town, four themes commonly appeared in young mother's narratives about becoming and being pregnant, and their experiences of motherhood. First, most young mothers said the pregnancy was unexpected. The surprise quickly turned to fear—fear of how the news of the pregnancy would be received by their parents or guardians, and by the genitor and his family. Parents or guardians often reacted with anger and would punish the pregnant teenager in some way. The genitor often denied paternity and refused to pay *inhlawulo*, which serves to acknowledge the child's paternity and clarifies who their ancestors are. Its successful negotiation has implications for care practices and for the performance of life‐course rituals. The payment (or even negotiation without full payment) of *inhlawulo* makes it possible for the child to be cared for by paternal kin, not just maternal kin. Second, young mothers revealed that the people who, according to general discourse, are supposed to provide them with knowledge about how to prevent pregnancy—their parents, teachers, and nurses—were not doing so. Third, young mothers told me they found out that they were pregnant in the second trimester. This was often because a family member accused them of being pregnant after observing the changes in their physical appearance. The late realization had implications for prenatal care and could lead to a more medicalized birth. Fourth, in their telling of their experience of teenage pregnancy, shame and regret were constant themes. Young mothers described how difficult it was to attend school. They spoke about the shame they were made to feel and their loneliness, especially when their families were unsupportive.

This was 25 years ago. Since 2016, I have noticed a change in how young mothers speak about their experiences. The discourse has moved from one of shame and regret to one of responsibility and reconfiguring care practices. Care for children in South Africa mostly happens within families. Families are varied, fluid, and flexible, and they change over time. As families change, so too do the configurations of care (Mkhwanazi & Manderson, 2020). It is my long‐term ethnographic fieldwork that has enabled me to notice and be attentive to these changes, both in discourse and in practice. Anthropology enables these insights. More importantly, anthropology has encouraged me to reflect on how larger structural changes—whether social, economic, or even policy‐related—affect individual lives, families, and communities. These considerations, for me, cannot but lead to a curiosity about the reproduction *of* and *in* families residing in urban Black townships.

Indeed, witnessing my young doctoral research interlocutors (aged 13 to 22 when I first met them) become mothers and fathers deepened my interest in the creation of “young families” and decisions about reproduction and caregiving in contexts in which teenage mothers and their mothers are both still of reproductive age. Paying attention to how the care of children is negotiated within and between families became important (on intergenerational fertility in the context of HIV, see Swartz, 2017). I also came to understand that the dynamics of the relationships of care within families, particularly between and within genders and generations, are complex. It brought to my attention how people's experiences and understandings of care and care work are dependent on their stage in the life course, and that this can differ radically between generations, partly because of the different ways that people's lives have been affected by apartheid or its legacy. This has been key in my thinking about the relationship between children, families, and the state in the context of negotiations around the care of children, whether within families (biological and foster) or in state institutions. Anthropology has been my vehicle to do this critical work.

It has not been easy. Such research takes a toll on one's body and emotions. Early on in my research, I learned very quickly that the ethnographic project was a visceral one, and that I needed to pay attention to the emotional aspects (good and bad) of my ethnographic encounters. Research changes us, often without our realizing it.

## ORPHANS OF THE LIVING

In 1999 I researched how children came to be seen as “in need of care.” The research was heartbreaking, but it was important. At the time, South Africa was revising the Child Care Act, and there was a need for research on how Black communities and families defined a child as “in need of care” and what recourse was followed to ensure that children were cared for. This was to see if there were any misalignments between local practices and the Child Care Act. According to the Child Care Act of 1983 and its subsequent amendments, a child in need of care is defined as one who (a) needs to be removed because of neglect and/or abuse; (b) has to appear in court because of the death or disappearance of a parent figure and in circumstances where there is no one to look after him or her; or (c) is described by current caregivers as uncontrollable because of his or her behavior (Skelton, 1998).

As part of the research, I spent time at Elukhuselweni, a “place of protection.”^3^ Children defined as “in need of care” were temporarily placed in a place of protection with the hope that they would find foster parents, adoptive parents, or that their family circumstances would change. At the time of my research, Elukhuselweni housed 46 children under the age of five.

The nursery, where the youngest children slept, had immaculate white walls and brightly colored cots arranged in precise rows facing each other. The order and stark cleanliness competed with the smell of babies and milk formula. I will never forget three children in particular—Ntombethu, Nkululeko, and Nkosingiphile—whose presence and interactions demonstrated care, and also highlighted the insidious, ongoing effect of apartheid in how children experience care and who provides that care.

On my first visit to the nursery, my attention was caught by the stocky figure of Ntombethu clinging to the red bars of her cot, staring at me. Our eyes met, and she stuck her tongue out. Immediately her face lit up as she smiled at her daring wit. Ntombethu was a year old and still unsteady on her feet. She and her brother, Simphiwe, were two of the children “in need of care” at Elukhuselweni: they had been removed by social workers from their mother's care because she was said to be “mentally unstable.” Despite being unable to physically care for her children, the mother frequently visited Elukhuselweni. Her act of care was her daily presence at the home.

On the same day, I also met one‐and‐a‐half‐year‐old Nkululeko, who was Ntombethu's playmate. He had been abandoned at a nearby hospital and was placed at Elukhuselweni by a social worker. Nkululeko's favorite game was to slip his feet into one of the childcare workers’ shoes and pace around the disinfected floor, concentrating on each step. His mannerisms often struck me as mature for his age. I would often catch him putting on baby mannerisms, which, it seemed, he was using to temper his maturity. Sometimes it seemed he wasn't sure how he should be acting, and he often took his cues from younger Ntombethu. He struck me as a very kind soul. When the toys were brought out at playtime, he was always willing to share his with Ntombethu. I often saw him happily step off the rocking horse to give Ntombethu a turn and even help her rock it since she had not yet learned how. Nkululeko was placed with foster parents after eight months of being at Elukhuselweni.

On April 29, 1999, a swelteringly hot day, Nkosingiphile arrived at Elukhuselweni. She was led in by one of the childcare workers who was carrying a cream‐colored pillowcase containing all her belongings. Wearing what looked like her Sunday best—a brightly colored dress with a long‐sleeved shirt underneath and miniature gum boots—she stood still in the middle of the room as everyone's eyes fell on her. Moments later, she walked toward the glass doors and looked outside at the other children playing in the sunshine. Tears welled up in her eyes and trickled down her cheeks. She began crying “Mummy” over and over. Later that day, she met some of the preschool children that she had seen playing outside. Under their scrutiny, she stayed apart on her own, alternating between crying and watching what was going on around her, wondering at the confusion of strangers. When bath time came, she hung back, not used to being one of the naked children waiting their turn to be soaped, rinsed, and dried. She called out for her mother again, and Sibusiso, one of her age‐mates, answered her with a copied “mummy.” They called and answered each other until they burst into laughter. He moved closer to her and held out his hand. She had made a friend. Nkosingiphile was living with HIV. She was unlikely to be adopted or taken into foster care because at the time HIV was shrouded in stigma. Her only hope was to wait for what could amount to years to get a place at Nazareth House, the only home for abandoned children living with HIV in Cape Town.

While conducting fieldwork for this research project, I found myself in a constant state of tearfulness. I was unprepared for the emotions that would surface. This was four years after South Africa had transitioned to democracy, but the specter of apartheid was still visible in these children's lives. In hindsight, this research planted the seeds for my deep‐rooted interest in studying life course, kinship, and care. I could see and recognize how my interlocutors, both adults and children, cared for and about each other. This would not have happened had I not been willing to spend time with, listen to, and feel that constant lump in my throat. When one conducts ethnographic research, it is almost impossible not to feel and care. Caring, I believe, makes us better anthropologists, and the good of ethnography is that it takes us away from the armchair or desk and into a place where life unfolds.

## THE ROOT OF FAMILIES AND CARE

While “care” and “the good” (Robbins, 2013) have become popular concepts to think about and with, translating this to research is not straightforward. As I pointed out at the start of the essay, practices that seem to be acts of care by anthropologists are not always seen as such by their interlocutors. In my overall ethnographic work, the practice of care that I have become attuned to is that of generational work. Care is subjective. Care work is the labor that helps others survive, thrive and sustain relationships. There are infinite ways of providing care, and correspondingly diverse ideas about what constitutes care and care work. Depending on the needs of the person being cared for and the ability of the caregiver, it can include activities as diverse as

growing, harvesting, purchasing and preparing food, cleaning and home maintenance, assisting with transport, medical appointments, liaising with government staff and others, and assisting with children with social interactions, as well as personal tasks such as lifting, carrying, washing, going to the toilet and feeding. (Mkhwanazi et al., 2018, p. 70)

The good of anthropology is that it enables us as anthropologists to tease out the connections and contestations of care and care work in context.

The majority of my research has focused on the experiences of young mothers. In South Africa, pregnancy and parenthood during the teenage years receive a lot of attention, both in the media and in academia. Most of this attention is negative and focuses specifically on poor, Black girls. My ethnographic project has been to shift this discourse from narratives of irresponsibility, of “an epidemic” of teenage pregnancy, and of young mothers being “unproductive citizens,” to one that shows what families have to do to ensure that a teenage pregnancy does not disrupt their already‐precarious lives (Mkhwanazi, 2017). This approach to teenage pregnancy has made me more aware of care—in particular the decision‐making about who provides care; the actual care work involved; and the variations in family composition that exist in South Africa as a consequence of caregiving decisions.

The young woman who shifted the way I understood the dynamics of teenage pregnancy and motherhood was 18‐year‐old Rethabile. When I met her in 2016, she was living with her mother and her 13‐year‐old sister. When she suspected that she was pregnant, she told her boyfriend, Mandla, and he assured her that he would support her. This was unusual in my earlier research from 1999 to 2002, as then the denial of paternity was widespread in Black communities (Ngabaza, 2011; Nkani, 2012)

The first person the couple told about the pregnancy was Rethabile's cousin; her mother was second. Rethabile told me she was disappointed with her mother's reaction (she had suggested a termination). Again, this was different from my earlier research. Many young mothers told me that although they had considered a termination, they did not go through with it because they were concerned that they would never be able to have children in the future. They also expressed how afraid they were of the stigma that would follow if people found out that they had terminated a pregnancy. Rethabile's mother had suggested a termination because she was concerned that Rethabile might drop out of school or that Mandla might leave her in the future. This was not an unwarranted concern.

Mandla's family acknowledged the pregnancy and supported the couple during the pregnancy and afterward, with childcare. This was again a significant change from earlier experiences of teenage motherhood, in which the denial of paternity was common and the genitor and his family were absolved from any care responsibility toward the child.

Rethabile described to me the birth of her daughter, Mbali, at Baragwanath Hospital. She said she had initially gone to the local clinic, but, because her blood pressure was high, the nurses told her to go to Baragwanath Hospital. On being advised to go to the tertiary hospital, Rethabile panicked because she had heard that the nurses at Baragwanath were negligent and that babies died as a result. She described the chaos and appalling conditions when she arrived at the maternity ward. Since the maternity ward was stretched beyond its capacity, she was sent to another ward in a different part of the hospital. She described her shock at entering an empty and clean ward. She was greeted by four midwives and a doctor. They attended to her, assuring her that she should relax and that everything would be all right. She described feeling relieved and happy that she had not had the bad experience she had been expecting from the rumors she had heard.

Mbali was diagnosed with jaundice. Since they did not have medical aid (health insurance), which would mean that they would have to pay exorbitant rates in a private hospital, Mbali spent the first week with her daughter in Baragwanath Hospital. Rethabile and her mother took turns sleeping at the hospital. After they left the hospital, Mbali went back to school, and both of her grandmothers helped look after the baby while accommodating their different work schedules and the young couple's education.

Rethabile's story, like many others that I have since heard, draws attention to the importance of providing care and support to young mothers. Her experience of pregnancy, childbirth, and motherhood was an affirmative one because she was supported and cared for by her own family, by the genitor's family, and by the birth practitioners. This made a great difference both in her transition to motherhood and in her future prospects. Rethabile's experience confirmed the importance of care and care work. It enabled me to see the importance of exploring the social relationships and communities of care that cohere around the advent of a teenage pregnancy rather than identifying its causes and consequences, which much of the research in South Africa still does. Her experience also pointed to the kinds of support systems that are available to young parents and how these work—and this is where anthropology excels!

Had I not continued my research with young mothers and extended this to young families, I would not have encountered this critical perspective on early motherhood. Looking at care is a more generative way to think about teenage pregnancy, motherhood, and young families. This care‐centered and longitudinal approach to early parenthood is unusual in South Africa. It is, I believe, what has brought my work to the attention of policymakers. This kind of impact is important, since the family remains one of the places where the legacy of apartheid is most visible, lingering and palpable.

Through anthropology, I have explored relationships of care between and across generations. When a teenager becomes a mother, the questions of care that arise concern not only care for the child but also care between generations of women—between daughters, mothers, and grandmothers. These forms of care, central to the well‐being of “young families,” are not always apparent or experienced positively. Looking at the body of my work—which speaks to these changes over time—I am proud of my archive of interviews, field notes, and diaries, which are a testament to the good of long‐term ethnographic research and anthropology.

## CARING FOR THE NEXT GENERATION—THE POTENTIAL GOOD OF ANTHROPOLOGY

Anthropology has, for a long time, been a field plagued with paradoxes, tensions, inequalities, and failures of care—for interlocutors, for training the future generations of anthropologists, and for the discipline—even amid intentions, aspirations, and performances of care. In South Africa, it took the student protests of 2015–16 for anthropologists to begin to shift their curricula to look to African people as worthy academics and to take seriously the work of Black African scholars. Anthropology students in South Africa are still being trained to value Western theory and literature written by scholars based outside the continent (Tella, 2020). This is because many of the people who hold positions in anthropology departments (including myself) received their PhDs from universities in the Global North, where what counts as the anthropological canon are not the works of African‐based scholars.

Anthropology is a discipline that now has at its core the ethic of care, which is more than just “do no harm.” This was not always the case. Nowadays, anthropologists endeavor to be with, sit with, listen to, converse with, and share in the everyday lives of their interlocutors. Furthermore, they try, as best as they can, to explain to others how their interlocutors make sense of their everyday lives and why they turn to certain courses of actions and not others.

Having been tethered to anthropology in South Africa, and having read many ethnographies about health, healing, and medical systems in Africa, I have realized that anthropology has not always been good at documenting and theorizing African lifeworlds and world‐making (Mkhwanazi, 2016). As we move forward, those of us who are anthropologists in and of Africa need to rethink the practice of anthropology on the continent so that we take seriously African lives, experiences, thinking, and dignity. Building my networks and collaborations with other anthropology departments on the African continent, I have been digging my heels deeper into anthropology. I believe that when practiced with care, anthropology holds the promise of helping us understand and theorize African world‐making, and perhaps to better understand care itself.

## ENDNOTES

1 Among those who studied at Fort Hare are the following: from South Africa, Z. K. Mathews (academic), D. T. T. Jabavu (academic), Oliver Tambo (president of the ANC), Nelson Mandela (first Black president of South Africa), Govan Mbeki (politician and anti‐apartheid activist), Robert Sobukwe (lawyer and president of the Pan Africanist Congress), and Mangosuthu Buthelezi (politician); from Kenya, Eliud Mathu (politician) and Charles Njonjo (lawyer and politician); from Zimbabwe, Robert Mugabe (second president of Zimbabwe) and Herbert Chitepo (lawyer and politician); as well as Sir Seretse Khama (first president of Botswana), Ntsu Mokhehle (third prime minister of Lesotho), and Yusuf Lule (fourth president of Uganda).

2 “Teenage Pregnancy Is Statutory Rape!,” fundraiser started by Kekeletso Khena, August 8, 2023, https://www.change.org/p/stop‐statutory‐rape‐in‐south‐africa.

3 Elukhuselweni is a pseudonym, as are the names of the research participants.
